# Neuron Derived Cold-Inducible RNA-Binding Protein Promotes NETs Formation to Exacerbate Brain Endothelial Barrier Disruption after Ischemic Stroke

**DOI:** 10.14336/AD.2024.0204-1

**Published:** 2024-02-04

**Authors:** Zhifang Li, Shuai Sun, Qinghui Xiao, Senwei Tan, Huijuan Jin, Bo Hu

**Affiliations:** Department of Neurology, Union Hospital, Tongji Medical College, Huazhong University of Science and Technology, Wuhan 430022, China.

**Keywords:** Acute ischemic stroke, brain endothelial barrier destruction, NETs formation, CIRP

## Abstract

In ischemic stroke, neutrophils are the first-line peripheral immune cells infiltrating the brain tissue to form neutrophil extracellular traps (NETs). The present study aimed to investigate the role of neuronal cold-inducible RNA-binding protein (CIRP) in promoting NETs-induced brain endothelial barrier destruction and cerebral edema after ischemic stroke. We found that the expression of NETs and neuronal CIRP in the penumbra increased at 6 hours after transient middle cerebral artery occlusion (tMCAO) and increased significantly at 24 hours, reaching a peak at 3 days. NETs degradation or CIRP inhibition can alleviate the leakage of brain endothelial barrier and reverse the decreased expression of tight junction proteins (zonula occludens-1, claudin-5 and occludin) in tMCAO mice. Oxygen-glucose deprivation/reperfusion treated primary neurons or recombinant CIRP could induce NETs formation via TLR4/p38 signaling pathway *in vitro.* Transcription factor specificity protein 1 (sp1) was responsible for the increased neuronal CIRP expression and the inhibition of sp1 could suppress the increased CIRP expression, reduce NETs formation, and diminish brain endothelial barrier leakage in tMCAO mice. We also found the upregulated CIRP level was associated with severe cerebral edema in patients with acute ischemic stroke. In conclusion, the increased expression of transcription factor sp1 after ischemic stroke can lead to elevated CIRP expression and release from the neurons, which subsequently interacts with neutrophils and promotes NETs formation, resulting in brain endothelial barrier destruction and cerebral edema.

## INTRODUCTION

Ischemic stroke has become the leading cause of disability around the world with limited treatment options, resulting in a huge public health burden [1]. Currently, rapid reperfusion by thrombolysis or thrombectomy remains the main therapeutic strategy, which is time-critical, and a great number of patients cannot benefit from reperfusion [2, 3]. Recent studies have suggested that malignant cerebral edema is the main cause of ineffective reperfusion [4, 5], which is largely attributed to the breakdown of brain endothelial barrier after reperfusion [6]. Thus, protecting brain endothelial barrier from damage is of significant importance to improve the prognosis of patients with acute ischemic stroke.

After ischemic stroke, various peripheral immune cells are activated and infiltrate into the ischemic brain [7]. Among these cells, neutrophils are considered as the first line responder, and play a detrimental role in brain endothelial barrier disruption by producing reactive oxygen species, proteases (such as matrix metalloproteinase, elastase, proteinase 3), and lipocalin-2 to degrade the brain endothelial barrier structure [8-10]. During neutrophil activation, the mixture of web-like chromatin, microbicidal agents, and proteins are released from neutrophils to form neutrophil extracellular traps (NETs) [11]. NETs have been reported to mediate the inflammatory responses and even lead to inflammatory storms in sepsis and atherosclerosis, resulting in devastating effects [12, 13]. Further, NETs contain several cytotoxic proteases such as elastase, as well as peroxidase, and pro-inflammatory mediators, which can damage the vascular endothelium and increase the vascular permeability [14]. Of note, the NETs content was significantly increased in the plasma of patients with ischemic stroke, which was closely related to the poor prognosis [15]. These results suggest that NETs formation may also occur after ischemic stroke, which can disrupt brain endothelial barrier integrity.

Cold-inducible RNA-binding protein (CIRP) was first discovered 20 years ago. The expression of CIRP can be induced by cold stimuli [16]. In the unstimulated state, CIRP mainly exists in the nucleus as an RNA-binding protein, mediating various biological processes [16]. However, several stimuli such as hypoxia and inflammation may lead to its translocation from nucleus to the cytoplasm and subsequent release from cells as damage-related molecular patterns (DAMPs) [16]. CIRP promoted NETs formation and mediated inflammation in an animal model of sepsis [12] and was associated with endothelial disruption in the lung [17]. Further, both the serum and tissue levels of CIRP were increased in hepatic and mesenteric ischemia-reperfusion, while down-regulation of CIRP alleviated the tissue damage, indicating the role of CIRP in ischemia-reperfusion injury [18, 19]. However, it remains unclear whether CIRP also participates in NETs formation after acute ischemic stroke.

In this study, we first investigated whether NETs formation was triggered after ischemic stroke leading to the early destruction of brain endothelial barrier. Next, we evaluated the expression and cellular localization of CIRP after ischemic stroke and investigated whether CIRP induced NETs formation in ischemic stroke. Finally, we explored the mechanism of regulation of CIRP expression and promotion of NETs formation after ischemic stroke, in order to identify a novel therapeutic target for clinical intervention and prognosis of patients with ischemic stroke.

## MATERIALS AND METHODS

### Establishment of the tMCAO model

**Figure 1.**
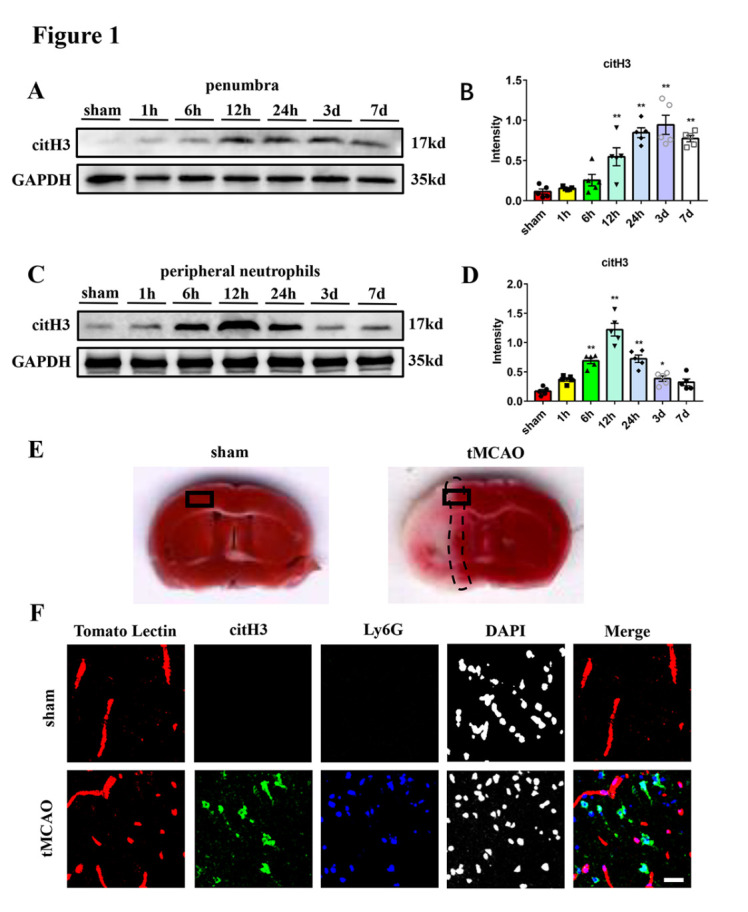
**Neutrophils form NETs in transient middle cerebral artery occlusion mice**. (A and B) Western blot analysis and statistical results of citH3 expression in the ischemic penumbra at different times after tMCAO (n = 5/group). Protein levels were normalized to the GAPDH signal. (C and D) Western blot analysis and statistical results of citH3 expression in the peripheral neutrophils at different times after tMCAO (n = 5/group). Protein levels were normalized to the GAPDH signal. (**E**) Illustration of the origin of the representative sham and tMCAO mice cerebral images (within the black frame). The area within the dotted line represents penumbra in the tMCAO brain. (**F**) Representative images of Tomato Lectin (red), citH3 (green) and Ly6G (blue) staining of mice brain sections from sham and tMCAO (24 hours) groups. DAPI (Grays) was used to stain nuclei. The merged image includes the stain of Tomato Lectin, citH3 and Ly6G. Scale bar: 25 μm. *p < 0.05, **p < 0.01. tMCAO, transient middle cerebral artery occlusion; citH3, citrullinated histone H3.

All mice experiments were approved by the Animal Care and Use Committee, Huazhong University of Science and Technology. Transient middle cerebral artery occlusion (tMCAO) was induced as previously described [20]. In brief, 6-8-week-old C57BL/6 mice were first anesthetized with isoflurane (Shenzhen Rayward Life Technology Co., Ltd) before the surgical procedure. Next, the skin on the neck was disinfected and a midline incision measuring about 1 cm was made. The subcutaneous tissue was separated to fully expose the right internal carotid artery (ICA), external carotid artery (ECA) and common carotid artery (CCA). A standardized 6-0 silicon-coated nylon filament (Yushun Biotech) was inserted into the ECA proximally bypassing the CCA and finally advanced via the ICA to occlude the entrance of the middle cerebral artery (MCA) until feeling the resistance. The mice remained anesthetic on the surgical plane for 1 hour of ischemia, and then the filament was withdrawn to allow full reperfusion of 1 hour, 6 hours, 12 hours, 24 hours, 3 days and 7 days depending on the experimental design. The stroke core refers to the tissue with completely absent perfusion, while ischemic penumbra is defined as the area where perfusion is decreased, but it still contains viable cells. As shown in Figure 1E, the area within the dotted line represents penumbra in the tMCAO brain and is used for penumbra collections. The sham group was subjected to the same procedures except for MCA occlusion. The area used for collecting the sham and tMCAO samples was shown in the black frames of Figure 1E.

### Drug treatments

To digest NETs, DNase I (1 mg/mL, Abcam) was injected intravenously into the tMCAO mice at a dose of 10 µL immediately after the filament was removed, followed by intraperitoneal injection of 50 μL. Twelve hours later, the tMCAO mice were intraperitoneally injected with another 50 μL of DNase I. The PAD inhibitor Cl-amidine (MedChemExpress) was intraperitoneally injected at 10 mg/kg immediately after the filament was removed. To inhibit CIRP, 200 μL of C23 (1 mg/mL, AtaGenix) was injected intravenously into tMCAO mice immediately after the filament was removed. To suppress sp1, mithramyin A (MMA, MedChemExpress) was dissolved in 10% dimethylsulfoxide (DMSO, Sigma-Aldrich) and intraperitoneally injected at 250 μg/kg after the filament was removed. Phosphate-buffered saline (PBS, Biosharp) was used as vehicle for the treatment of DNase I, Cl-amidine and C23, while 10% DMSO was used as vehicle for the treatment of MMA.

In the cellular experiment, recombinant murine CIRP (rmCIRP) was purchased from AtaGenix and added at concentrations of 1, 2.5, and 5 µg/mL for 6 hours to stimulate NETs formation *in vitro*. The resatorvid (MedChemExpress), PD169316 (MedChemExpress), C23 (AtaGenix) and MMA (MedChemExpress) was added at the concentration of 100 nM, 10 µM, 0.3 µg/mL, and 300 nM, respectively.

### Evan’s blue extravasation

To evaluate the brain endothelial barrier disruption, Evan’s blue extravasation was carried out 24 hours after tMCAO. The tMCAO mice were injected with 4% Evan’s blue dye (4 mL/kg, Sigma-Aldrich) via the tail vein. Sham mice were injected with the same dose of Evan’s blue dye. Four hours after circulation, the mice were anesthetized and perfused with PBS to dissert the brain tissue. The hemisphere samples were homogenized with dimethylformamide and incubated at 55°C for 24 hours to extract Evan’s blue dye. After centrifugation at 10,000 × g for 20 minutes, the supernatant was collected and measured at 620 nm using a microplate reader to determine the concentration of Evan’s blue dye.

### Detection of Extravascular Dextran

To detect the extravasation of dextran, the FITC-labeled dextran (40 kDa, Sigma-Aldrich) was used. 24 hours after tMCAO, the mice were intravenously injected FITC-dextran at a concentration of 50 mg/mL circulating for 5 minutes. Then, the mice were sacrificed, and the brains were removed into 4% paraformaldehyde solution (Biosharp) at 4 °C overnight and subsequent 30% sucrose (Biosharp) solution at 4 °C for 48 hours. A vibratome was used to cut the brains into coronal sections (8 μm thick). Thereafter, the brain slices were visualized on a TCS SP5 multiphoton laser scanning confocal microscope (Nikon, Tokyo, Japan).

### Viral injection

The Adeno-associated virus (AAV) 2/9 serotype (pAAV-hSyn-EGFP-3Xflag-miR30shRNA-cirp, Obio Technology, Shanghai, China) was used to specifically downregulate neuronal CIRP expression and null vectors (pAAV-hSyn-EGFP-3Xflag-miR30shRNA-NC, Obio Technology, Shanghai, China) were injected as control. In brief, the online design tool was applied to predict the targeting sequences of the mouse CIRP gene. Then, the synthesized sequences were constructed into double-enzymatic linearized target vectors and packaged into viruses. Four weeks before tMCAO, the mice were inhaled with 2% isoflurane to induce anesthesia and fixed on the stereotaxic frames. AAV solution (1 × 10^^13^ µg/mL) was injected into the intracerebroventricular space at a rate of 0.05 μL/min to a total volume of 2 μL. The coordinate of the intracerebroventricular site was as follows: Anterior-Posterior (AP), 0 mm; Medial-Lateral (ML), 1 mm; Dorsal-Ventral (DV), 1.5 mm.

### Primary mouse cortical neuronal culture

To obtain the primary cortical neuron culture, E17-18 fetuses were isolated from pregnant C57BL/6 mice and incubated in ice-cold DMEM/F12 medium (Cytiva). Next, the fetal brains were removed and placed in the dissecting medium: 50% DMEM/F12 + 50% D-hanx (Biosharp). The cortical tissue was separated using microscopic forceps and incubated in the digesting medium containing 0.125% trypsin (Biosharp) and 0.1 mg/mL DNase (Biosharp) in the 37°C incubator for 15 minutes. The cells were pelleted and centrifuged at 1000 rpm for 5 minutes. The digested cells were resuspended with the neuronal medium: serum-free Neurobasal (Gibco) + B27 (2%, Gibco) + penicillin-streptomycin (1%, Biosharp) solution, and then seeded in culture flasks precoated with poly-l-lysine (100 µg/mL, Biosharp) for further studies, replacing half of the medium every 3 days.

### Oxygen-glucose deprivation/reperfusion (OGD/R)

To simulate the ischemia and reperfusion conditions *in vitro*, the primary neurons were exposed to OGD/R. Briefly, after washing twice with sterile PBS, primary neurons were cultured in glucose-free DMEM medium (Cytiva). The primary neurons were placed in an anaerobic incubator (37°C, 5% CO_2_, 1% O_2_ and 94% N_2_) for 2 hours. Subsequently, primary neurons were reoxygenated and incubated under normal culture conditions for indicated time in subsequent experiments.

### Isolation of peripheral neutrophils

In this study, peripheral neutrophils were isolated from mouse blood using a mouse peripheral blood neutrophil isolation kit (LZS1100, Tianjin Haoyang Biological Manufacture Co., LTD.). Briefly, blood was collected from anesthetized tMCAO mice in sterile silicone tubes containing anticoagulant (TBDTM-0050, Tianjin Haoyang Biological Manufacture Co., LTD.) at a 1:1 anticoagulant-to-whole blood volume ratio. Another centrifuge tube was filled with 3 mL of separation solution and 1.5 mL of 80% separation solution. The anticoagulant blood in the sterile silicone tube was mixed with the erythrocyte sedimentation solution at a ratio of 1:1. The mixed blood was slowly added to the top of 80% separation solution and centrifuged at 800 × g for 20 minutes. Two distinct cell layers were detected after centrifugation, with the lower layer containing neutrophils. The neutrophils were collected and washed twice using the cleaning solution. The isolated neutrophils were resuspended with the neutrophil medium: RPMI-1640 (Cytiva) + 10% fetal bovine serum (Gibco) + 1% penicillin-streptomycin solution (Biosharp) and seeded on plates for further studies.

### CCK-8 assay

A Cell Counting Kit-8 (Biosharp) was used to evaluate cell viability. In brief, 20 µL of CCK-8 solution was added into each cell culture well with 200 µl medium solution, incubating for 2 hours at 37 °C. Then, the microplate reader was used to measure the absorbance at 450nm.

### Dual-luciferase assay

The HEK293T cells were co-transfected with the CIRP luciferase reporter plasmid, sp1 plasmid and Renilla control reporter plasmid for 48 hours. Then, the HEK293T cells were harvested. The enzymatic activity of luciferase and Renilla were determined using the Dual Luciferase Reporter Assay Kit (Vazyme).

### Quantitative real-time PCR

The total RNA was extracted from brain tissue using the TRIzol reagent (Enzyme) following the manufacturer's instructions. After isolation, RNA was reverse-transcribed into cDNA using the PrimeScript™ RT Master Mix (Enzyme). The SYBR Premix Ex Taq II solution (Enzyme) was used for cDNA amplification and quantification. The thermocycler (Bio-Rad, Hercules, CA, USA) was used to perform quantitative real-time PCR. The primers for CIRP were 5′-GAGCAGGTCT TCTCCAAGTATG -3′ (forward) and 5′-GTCCACAGA CTTCCCATTCATA -3′ (reverse). The primers for TLR4 were 5′-GAGCCGGAAGGTTATTGTGGTAGTG-3′ (forward) and 5′-AGGACAATGAAGATGATGCCA GAGC -3′ (reverse). The primers for TREM1 were 5′- ACAACTACAACCCGATCCCTACCC -3′ (forward) and 5′-CAGGCTCTTGCTGAGAAGTCCAC -3′ (reverse). The primers for actin were 5′- GTGACGTTGA CATCCGTAAAGA-3′ (forward) and 5′- GCCGGACTC ATC GTACTCC -3′ (reverse).

### Western blot

Brain tissues or cell cultures were lysed in RIPA buffer (Beyotime Biotechnology) supplemented with 2% protease inhibitor cocktail (Beyotime Biotechnology) and 1% PMSF (Beyotime Biotechnology) for total protein extraction. For the isolation of lysosomal compartment, we used a kit from Thermo Scientific. Then, the protein samples were loaded onto the SDS-PAGE gel (Beyotime Biotechnology) in equal amounts for electrophoresis and the separated proteins were transferred to the polyvinylidene fluoride membranes (Millipore). After blocking with 5% skim milk (Beyotime Biotechnology) for 1 hour, the membranes were incubated with different primary antibodies (anti-citH3, 1:1000, ab5103, Abcam; anti-CIRP, 1:1000, 10209-2-AP, Proteintech; anti- zonula occludens-1 (ZO-1), 1:1000, 21773-1-AP, Proteintech; anti-Claudin-5, 1:500, A10207, Abclonal; anti-Occludin, 1:1000, 13409-1-AP, Proteintech; anti-p-p38, 1:1000, 4511, Cell Signaling Technology; anti-p38, 1:1000, 8690, Cell Signaling Technology; anti-sp1, 1:1000, 21962-1-AP, Proteintech; anti-Cathepsin D, 1:800, A19680, Abclonal; anti-GAPDH, 1:1000, AC001, Proteintech; anti-β-actin, 1:1000, AC026, Abclonal; anti-tubulin, 1:1000, M20005F, Abmart) at 4°C overnight, followed by corresponding secondary antibodies (HRP Goat Anti-Rabbit IgG, AS014; HRP Goat Anti-Mouse IgG, AS003). β-Actin, GAPDH or tubulin was used as the internal reference’s standards for total protein. Cathepsin D was used as the internal reference’s standards for lysosomal protein. Image J software (NIH) was used to measure the band intensity.

### Immunofluorescence

Immunofluorescence was performed as previously described [20]. In brief, the mice were perfused with PBS (Biosharp) and the brains were dissected. Then, the brains were fixed by 4% paraformaldehyde (Biosharp) at 4 °C overnight, immersed in 30% sucrose (Biosharp) at 4 °C for 48 hours and cut into 8 μm sections using a vibratome. After tissue antigen retrieval with citric acid buffer (Biosharp), the brain slices were blocked using 10% donkey serum (Biosharp) and 0.3% TritonX-100 (Biosharp) for 1 hour at room temperature. Isotype IgG antibody controls (1:50, AC042, Abclonal), secondary antibody only controls, and tissue minus/plus controls were applied to validate antibody specificity and distinguish genuine target staining from background before experiment. Then, samples were incubated with the primary antibodies: anti-citH3, 1:50, ab5103, Abcam; anti-Ly6G, 1:50, ab25377, Abcam; anti-CIRP, 1:100, 10209-2-AP, Proteintech; anti-NEUN, 1:50, ab104224, Abcam; anti-Iba-1, 1:50, ab5076, Abcam; anti-GFAP, 1:50, ab4674, Abcam; anti-Lycopersicon Esculentum (Tomato) Lectin, 1:50, DL-1177, Invitrogen; anti-Fibrinogen, 1:50, ab118533, Abcam; anti-MPO, 1:50, ab208670, Abcam; and anti-sp1, 1:100, 21962-1-AP, Proteintech. Finally, fluorescence-labeled secondary antibodies (Donkey anti-Rabbit IgG Plus 594, A32754, Invitrogen; Donkey anti-Rabbit IgG Plus 488, A21206, Invitrogen; Donkey anti-Rabbit IgG Plus 647, A31573, Invitrogen; Donkey anti-Sheep IgG Plus 488, A11015, Invitrogen; Donkey anti-Rat IgG Plus 488, A21208, Invitrogen; Donkey anti-Mouse IgG Plus 594, A21203, Invitrogen; Donkey anti-Goat IgG Plus 488, A11008, Invitrogen; Donkey anti-Chicken IgG Plus 488, A78948, Invitrogen) and DAPI were incubated. The brain slices were visualized on a TCS SP5 multiphoton laser scanning confocal microscope (Nikon, Tokyo, Japan).

### Enzyme-linked immunosorbent assay (ELISA)

The experiment measuring the concentration of serum and supernatant CIRP was performed using the ELISA kit (Bioswamp) according to the manufacturer’s instructions. To be brief, 50 μL of pre-diluted standard protein or sample protein were added into the standard protein or sample well, respectively. Then, 10 μL of CIRP antibody labeled with horseradish catalase was added into each well and incubated at 37°C for 30 minutes. Next, the reaction substrates A and B were mixed in a 1:1 ratio and added into each well to obtain a volume of 100 μL and incubated at 37°C for 10 minutes. Subsequently, 50 μL of stop solution was added, and the optical density (OD) of each well was read using a microplate reader.

**Figure 2.**
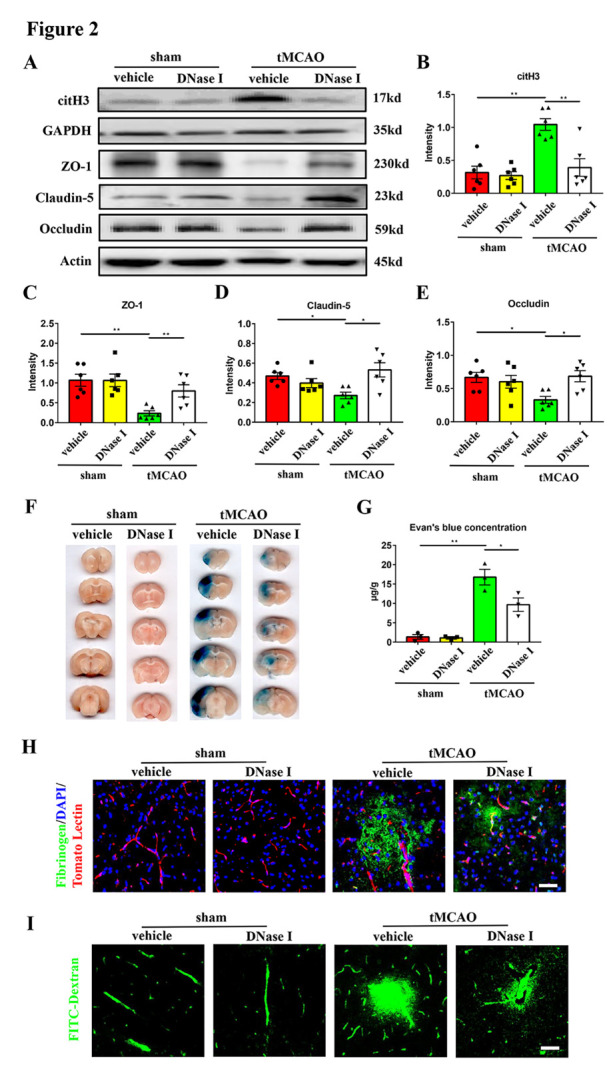
**DNase I treatment can attenuate brain endothelial barrier destruction in transient middle cerebral artery occlusion mice**. (**A-E**) Western blot analysis and statistical results of the expression of citH3, ZO-1, claudin-5, and occludin in the ischemic penumbra 24 hours after tMCAO (n = 6/group). Protein levels were normalized to the GAPDH signal for citH3 and actin signal for ZO-1, claudin-5, and occludin. (**F**) The Evan’s blue extravasation of mice brains in the coronal sections from sham + vehicle, sham + DNase I, tMCAO + vehicle and tMCAO + DNase I groups. (**G**) The Evan’s blue concentration expressed as μg/g of brain tissue (n = 3/group). (**H**) Immunofluorescence staining showing the leakage of fibrinogen (green) in the mice brains from these 4 groups. The blood vessels were stained with Tomato Lectin (red). DAPI (blue) was used to stain nuclei. Scale bar: 50 μm. I: Representative images of extravascular FITC-dextran fluorescence in the mice brains from these 4 groups. Scale bar: 50 μm. *p < 0.05, **p < 0.01. tMCAO, transient middle cerebral artery occlusion; ZO-1, zona occludens-1; citH3, citrullinated histone H3.

### Patients, Ethics and Data Collection

All study patients were recruited from January 1, 2023 to April 30, 2023 at Union Hospital, Tongji Medical College, Huazhong University of Science and Technology, Wuhan, China based on the inclusion criteria: (1) first-time diagnosis of acute ischemic stroke according to the guidelines [21]; (2) age above 18 years; and (3) stroke onset within 3 days. Patients who had (1) intravenous or intra-arterial thrombolysis; (2) mechanical thrombectomy; (3) malignant tumors; and (4) infections were excluded.

The study was conducted according to the Declaration of Helsinki and approved by the ethics committee of Tongji Medical College, Huazhong University of Science and Technology, Wuhan, China. All participants signed the consent forms and were informed of the study before inclusion.

The following clinical data were collected from the medical records: age, sex, National Institutes of Health Stroke Scale (NIHSS) score, smoking history, alcohol history, and medical history (Table 1). Based on magnetic resonance imaging (MRI) results, cerebral edema was classified into 3 subtypes: cerebral edema-1 (brain edema less than 1/3^rd^ of the hemisphere), cerebral edema-2 (brain edema greater than 1/3^rd^ of the hemisphere) and cerebral edema-3 (brain edema causing midline shift) [22]. Patients with cerebral edema-1 and -2 were included in the mild cerebral edema group and patients with cerebral edema-3 belonged to the severe cerebral edema group.

### Statistical analysis

GraphPad Prism 8 was used for data statistics, and all values were expressed as mean ± SEM. The distribution of data was first evaluated using the Shapiro-Wilk test. For data that did not conform to Gaussian distribution, non-parametric analysis was performed using the Mann-Whitney test for 2 groups or Kruskal-Wallis test for more than 2 groups. For data conforming to Gaussian distributions, statistical analysis of 2 groups was performed using unpaired Student’s t-test (unpaired Student’s t-test with Welch’s correction was used if their variances were unequal) and statistical analysis of more than 2 groups was performed using one- or two-way ANOVA followed by a Tukey’s post hoc test (Brown-Forsythe and Welch ANOVA test was used if their variances were unequal). The *p* value less than 0.05 was considered statistically significant.

**Figure 3.**
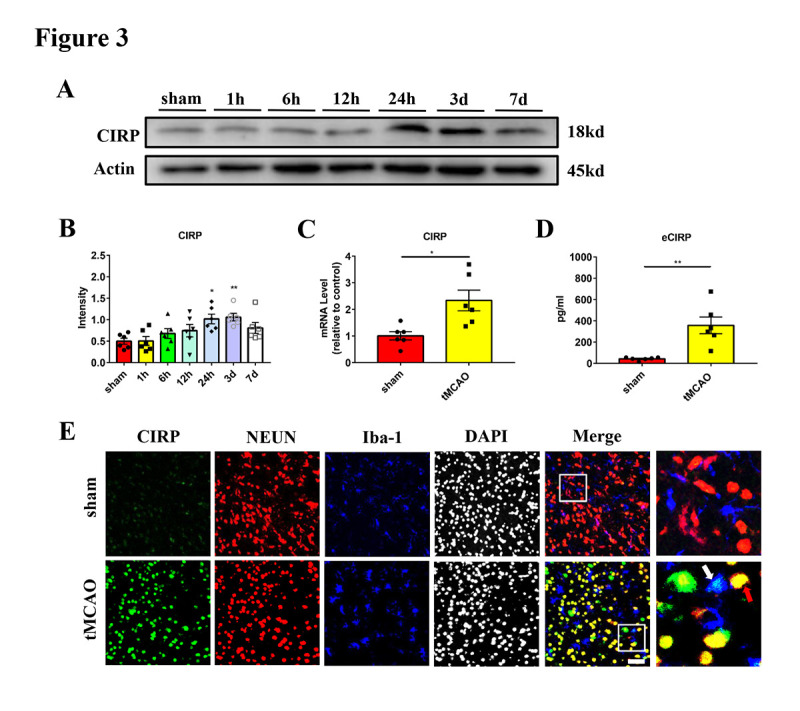
**The expression of CIRP was increased in neurons in transient middle cerebral artery occlusion mice**. (A and B) Western blot analysis and statistical results of CIRP expression in the ischemic penumbra at different times after tMCAO (n = 6/group). Protein levels were normalized to the actin signal. (**C**) Quantification of CIRP mRNA expression in the sham and tMCAO (24 hours) mice (n = 6/group). (**D**) Quantification of serum CIRP concentration in the sham and tMCAO (24 hours) mice (n = 6/group). (**E**) Representative images of CIRP (green), NEUN (red) and Iba-1 (blue) staining of mice brain sections from sham and tMCAO (24 hours) mice. DAPI (grays) was used to stain nuclei. The merged image includes the stain of CIRP, NEUN and Iba-1. The red arrow indicates the co-localization of CIRP and NEUN. The white arrow indicates the co-localization of CIRP and Iba-1. Scale bar: 50 μm. *p < 0.05, **p < 0.01. CIRP, cold-inducible RNA-binding protein; tMCAO, transient middle cerebral artery occlusion.

**Figure 4.**
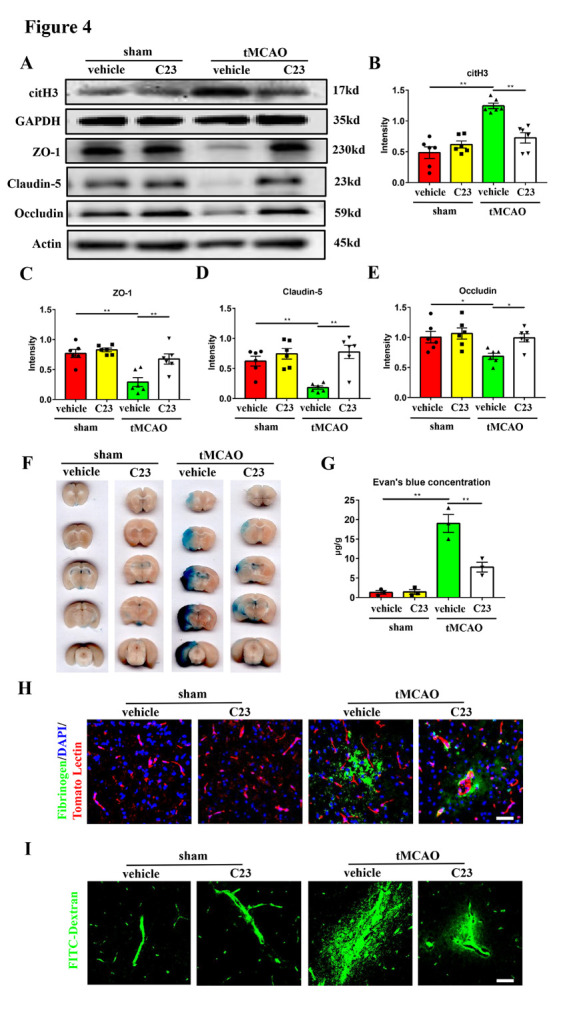
**CIRP inhibitor C23 can ameliorate NETs formation and brain endothelial barrier disruption in the transient middle cerebral artery occlusion mice**. (**A-E**) Western blot analysis and statistical results of the expression of citH3, ZO-1, claudin-5, and occludin in the ischemic penumbra 24 hours after tMCAO (n = 6/group). Protein levels were normalized to the GAPDH signal for citH3 and actin signal for ZO-1, claudin-5, and occludin. (**F**) The Evan’s blue extravasation of mice brains in the coronal sections from sham + vehicle, sham + C23, tMCAO + vehicle, tMCAO + C23 groups. (**G**) The Evan’s blue concentration expressed as μg/g of brain tissue (n = 3/group). (**H**) Immunofluorescence staining shows the leakage of fibrinogen (green) in the mice brains from these 4 groups. The blood vessels were stained with Tomato Lectin (red). DAPI (blue) was used to stain nuclei. Scale bar: 50 μm. (**I**) Representative images of extravascular FITC-dextran fluorescence in the mice brains from these 4 groups. Scale bar: 50 μm. *p < 0.05, **p < 0.01. tMCAO, transient middle cerebral artery occlusion; ZO-1, zona occludens-1; citH3, citrullinated histone H3.

## RESULTS

### NETs formation exacerbates brain endothelial barrier disruption in tMCAO mice

To explore whether neutrophils infiltrating the brain parenchyma form NETs after acute ischemic stroke, western blot was used to evaluate the expression of the NETs in the ischemic penumbra in the mouse model of tMCAO. We found that NETs expression started to increase at 6 hours post-reperfusion, with a significant increase at 24 hours, reaching a peak at 3 days in the ischemic penumbra (Fig. 1A-B). In addition, the peripheral neutrophils also formed NETs after tMCAO, and the expression of citH3 in the peripheral neutrophils began to increase as soon as 1 hours after reperfusion, reached the peak at 12 hours, and started to decrease thereafter (Fig. 1C-D). Further immunofluorescence staining confirmed that citH3, the marker for NETs, colocalized with the neutrophil marker Ly6G, and both the number of citH3^+^ neutrophils and the fluorescence intensity of citH3 were increased 24 hours after tMCAO (Fig. 1E-F). Moreover, most of the citH3^+^ neutrophils appeared in the brain parenchyma rather than the intravascular space (Fig. 1E-F). These results suggested that NETs formation mainly occurs in neutrophils infiltrating the brain parenchyma after tMCAO.

**Figure 5.**
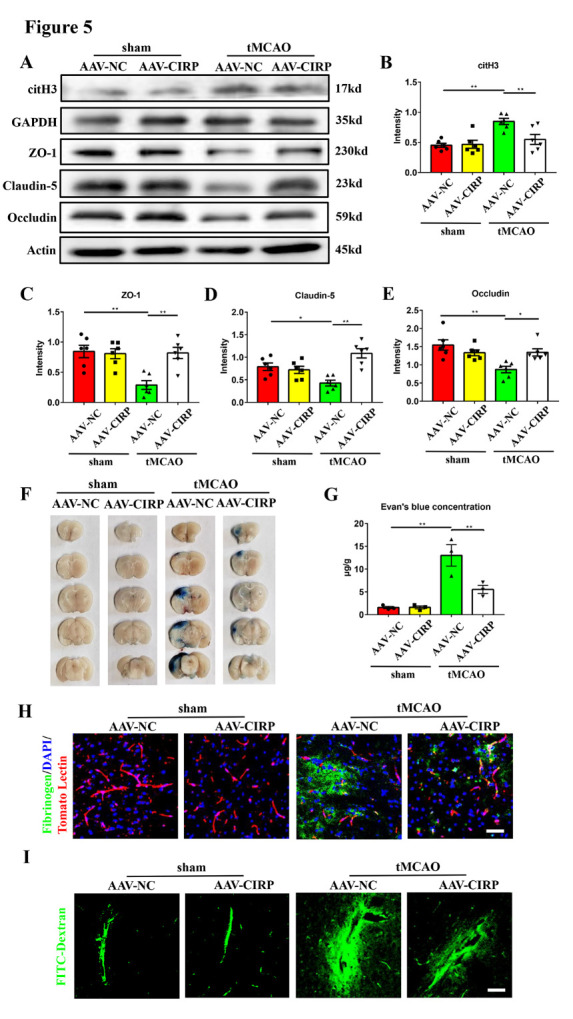
**Neuronal CIRP downregulation ameliorates NETs formation and brain endothelial barrier disruption in transient middle cerebral artery occlusion mice**. (**A-E**) Western blot analysis and statistical results of the expression of citH3, ZO-1, claudin-5, and occludin in the ischemic penumbra 24 hours after tMCAO (n = 6/group). Protein levels were normalized to the GAPDH signal for citH3 and actin signal for ZO-1, claudin-5, and occludin. (**F**) The Evan’s blue extravasation of mice brains in the coronal sections from sham+ AAV-NC, sham + AAV-CIRP, tMCAO + AAV-NC, tMCAO + AAV-CIRP groups. (**G**) The Evan’s blue concentration expressed as μg/g of brain tissue (n = 3/group). (**H**) Immunofluorescence staining shows the leakage of fibrinogen (green) in the mice brains from these 4 groups. The blood vessels were stained with Tomato Lectin (red). DAPI (blue) was used to stain nuclei. Scale bar: 50 μm. I: Representative images of extravascular FITC-dextran fluorescence in the mice brains from these 4 groups. Scale bar: 50 μm. *p < 0.05, **p < 0.01. CIRP, cold-inducible RNA-binding protein; tMCAO, transient middle cerebral artery occlusion; AAV, adeno-associated virus; ZO-1, zona occludens-1; citH3, citrullinated histone H3.

The breakdown of brain endothelial barrier integrity occurs at approximately 1 to 3 days after ischemic stroke, which coincides with the increased level of NETs in our study. To investigate whether modulating NETs can attenuate brain endothelial barrier destruction, we intravenously and intraperitoneally injected DNase I, which specifically degrades NETs, into the tMCAO mice immediately after reperfusion. The mice were randomly divided into four groups: sham mice + vehicle injection (sham + vehicle), sham mice + DNase I injection (sham + DNase I), tMCAO mice + vehicle injection (tMCAO + vehicle), and tMCAO mice + DNase I injection (tMCAO + DNase I). The brain endothelial barrier leakage was evaluated 24 hours after tMCAO. We found a significant decrease in NETs expression in the tMCAO + DNase I group compared with the tMCAO + vehicle group, indicating that DNase I successfully degraded NETs in the ischemic brain (Fig. 2A-B). In addition, western blot analysis showed that compared with the sham + vehicle group, the expression of tight junction proteins (ZO-1, claudin-5, and occludin) was significantly decreased in the tMCAO + vehicle group, while DNase I treatment abrogated this effect after 24 hours of tMCAO (Fig. 2A, 2C-E). Further, the tMCAO + vehicle group showed increased leakage of Evan’s blue, fibrinogen and FITC-Dextran in the ischemic cortex compared with the sham + vehicle group, while DNase I administration alleviated this leakage (Fig. 2F-I). Of note, the sham + DNase I group showed no difference compared with the sham + vehicle group in both expression of tight junction proteins and the leakage of brain endothelial barrier (Fig. 2A-I), which means that the DNase I cannot affect the permeability of brain endothelial barrier in normal mice. To further confirm the effect of NETs on leakage, we next treated the tMCAO mice with Cl-amidine, the inhibitor of peptidylarginine deiminase 4 (PAD4) which plays a crucial role in NETs formation [23]. In line with DNase I treatment, we found that the expression of tight junction proteins was also increased (Supplementary Fig. 1A-D) and the leakage of Evan’s blue, fibrinogen and FITC-Dextran was ameliorated (Supplementary Fig. 1E-H) in the Cl-amidine group after 24 hours of tMCAO. Taking together, these results suggested that NETs formation plays an important role in brain endothelial barrier destruction in tMCAO mice.

**Figure 6.**
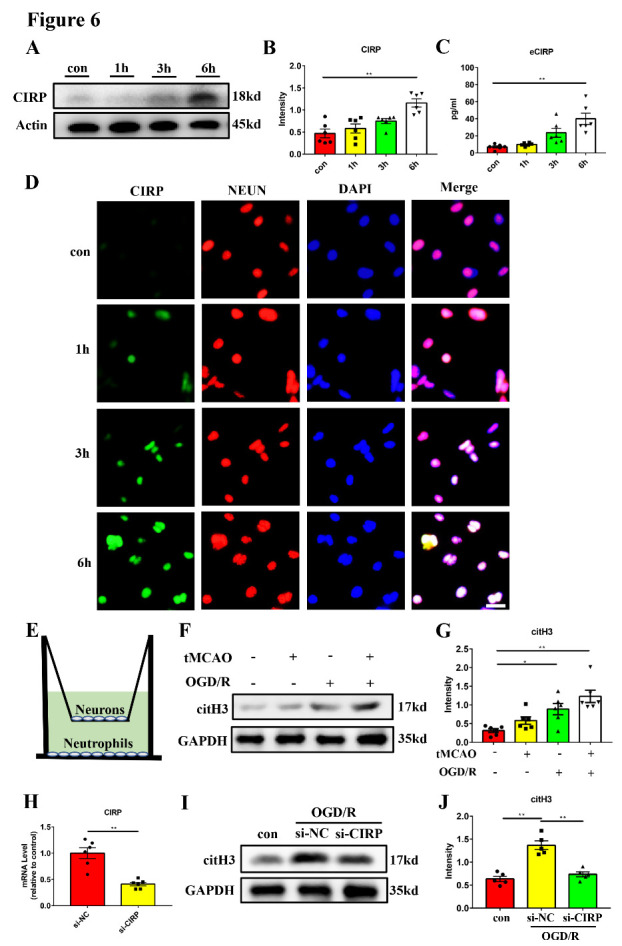
**Oxygen-glucose deprivation/ reperfusion stimulated primary neurons promote NETs formation by releasing CIRP *in vitro***. (A and B) Western blot analysis and statistical results of the expression of CIRP in primary neurons after different time of OGD/R (n = 6/group). Protein levels were normalized to the actin signal. (**C**) Quantification of CIRP concentration in the supernatant of OGD/R-treated primary neurons (n = 6/group). (**D**) Representative images of CIRP (green) and NEUN (red) staining of OGD/R-treated primary neurons. DAPI (blue) was used to stain nuclei. Scar bar: 25 μm. (**E**) The illustration of co-culture system of neutrophils and primary neurons. (F and G) Western blot analysis and statistical results of the citH3 levels in neutrophils under different conditions for 6 hours (n = 6/group). Protein levels were normalized to the GAPDH signal. (**H**) Quantification of CIRP mRNA expression in the si-NC and si-CIRP treated primary neurons (n = 6/group). (I and J) Western blot analysis and statistical results of the levels of citH3 in neutrophils co-cultured with si-CIRP pretreated primary neurons under OGD/R condition for 6 hours (n = 5/group). Protein levels were normalized to the GAPDH signal. Con, control group in which neutrophils were co-cultured with normoxia neurons; si-NC, siRNA of negative control; si-CIRP, siRNA targeting CIRP. *p < 0.05, **p < 0.01. OGD/R, oxygen-glucose deprivation/reperfusion; CIRP, cold-inducible RNA-binding protein; citH3, citrullinated histone H3.

### Ischemic neurons induce NETs formation by secreting CIRP in tMCAO mice

Previous studies have shown that NETs formation can be induced by CIRP in lungs of sepsis mice [24]. In addition, increased CIRP expression has been reported in mice intestinal and renal ischemia-reperfusion models [19, 25]. These results suggested that CIRP may also contribute to the occurrence of NETs after cerebral ischemic stroke.

In this study, we used the mouse tMCAO model to analyze CIRP expression in the ischemic penumbra. Western blot analysis showed that CIRP expression started to increase at 6 hours after tMCAO, significantly increasing at 24 hours, and peaking at 3 days (Fig. 3A-B). The CIRP mRNA level in penumbra and extracellular CIRP (eCIRP) level in serum were also significantly increased 24 hours after tMCAO compared with the sham group (Fig. 3C-D). In order to further elucidate the cellular expression of CIRP, we co-stained CIRP with markers of neuron (NEUN) and microglia (Iba-1), which are reported to be the main source of CIRP. We found that CIRP was mainly localized in neurons and its expression was also increased 24 hours after tMCAO (Fig. 3E). On the contrary, there were only a few CIRP^+^ microglia in the tMCAO group (Fig. 3E). To further validate the cellular expression of CIRP, we co-stained CIRP with endothelial cells (Tomato Lectin) or astrocytes (GFAP) and found only a few co-localizations (Supplementary fig. 2A-B). These results indicated that CIRP expression in neurons was increased in the ischemic penumbra after tMCAO in mice.

In order to determine whether the inhibition of CIRP can reduce NETs formation and brain endothelial barrier destruction, the tMCAO mice were intravenously injected with C23, a CIRP inhibitor, immediately after reperfusion. Mice were randomly divided into four groups: sham mice + vehicle injection (sham + vehicle), sham mice + C23 injection (sham + C23), tMCAO mice + vehicle injection (tMCAO + vehicle), tMCAO mice + C23 injection (tMCAO + C23). The brain endothelial barrier leakage was evaluated 24 hours after tMCAO. We found that the NETs expression in tMCAO + C23 group was lower than tMCAO + vehicle group (Fig. 4A-B), which suggested that inhibition of CIRP reduced the occurrence of NETs formation. Further, the tMCAO + C23 group showed higher expression of tight junction proteins than tMCAO + vehicle group 24 hours after tMCAO (Fig. 4A, 4C-E). It was further shown that the leakage of Evan’s blue, fibrinogen, and FITC-Dextran in the tMCAO + C23 group was significantly alleviated compared with the tMCAO + vehicle group (Fig. 4F-I). Significantly, the expression of tight junction proteins and the leakage of brain endothelial barrier in the sham + C23 group showed no difference compared with the sham + vehicle group (Fig. 4A-I), which means that the C23 cannot affect the permeability of brain endothelial barrier in normal mice. These results indicated that C23 can decrease NETs formation and brain endothelial barrier destruction in the mouse tMCAO model.

To further validate the role of neuronal CIRP in the induction of NETs formation and brain endothelial barrier destruction, we microinjected mice with AAV with neuron-specific promotor (AAV-CIRP) to specifically downregulate CIRP using stereotaxic frames. AAV which encoded the same neuron-specific promotor but did not influence CIRP expression (AAV-NC) was used as the negative control. The brain endothelial barrier leakage was evaluated 24 hours after tMCAO. Four weeks after injection, we found that the AAV mainly infected neurons in the brain (Supplementary fig. 3A) and neuronal CIRP expression was successfully decreased (Supplementary fig. 3B-C). To determine whether CIRP downregulation was sufficient to inhibit NETs formation in tMCAO mice, we measured citH3 level in the parenchyma and found that compared with the tMCAO + AAV-NC mice, citH3 expression was significantly decreased in the tMCAO + AAV-CIRP mice (Fig. 5A-B). CIRP downregulation significantly mitigated the decreased expression of tight junction proteins in mice after 24 hours of tMCAO (Fig. 5A, 5C-E). Compared with tMCAO + AAV-NC mice, the Evan’s blue, fibrinogen, and FITC-Dextran leakage was ameliorated in the tMCAO + AAV-CIRP mice (Fig. 5F-I). Our data showed that neuronal CIRP downregulation alleviated both NETs formation and brain endothelial barrier leakage in tMCAO mice.

### OGD/R-treated primary neurons promote NETs formation by releasing CIRP in vitro

To further confirm that neurons can increase the expression and release of CIRP after ischemic stroke, primary neurons were cultured in vitro and stimulated with OGD/R. The neuron viability after OGD/R treatment was detected by the CCK-8 assay (Supplementary Fig. 4A). The expression of CIRP in primary neurons was detected by western blot and immunofluorescence staining and the release of eCIRP in the supernatant medium was detected by ELISA. We found that primary neurons showed elevated CIRP expression 6 hours after OGD/R compared with control group (Fig. 6A-B and 6D, Supplementary Fig. 4B). ELISA assay also validated the significant increase of eCIRP concentration in the supernatant 6 hours after OGD/R (Fig. 6C). Since CIRP protein sequence lacks the secretion leader signal which is essential for the classic endoplasmic reticulum-Golgi-dependent secretion pathway [26], we next isolated the lysosomal compartment and examined whether neurons can release CIRP through the lysosomal secretion pathway. Compared with the control group, the lysosomal CIRP level was significantly elevated 6 hours after OGD/R, indicating the possibility of lysosomal secretion (Supplementary Fig. 4C-D). These results suggested that OGD/R induced the expression and release of CIRP from primary neurons.

Next, we established a co-culture system of primary neurons and periphery neutrophils in vitro to comprehensively analyze their interaction. As shown in Fig. 6E, normal neurons or neurons exposed to the OGD/R stimulus were seeded on the upper chamber, while peripheral neutrophils isolated from sham or tMCAO mice were co-cultured in the substratum culture plate for 6 hours. The protein level of citH3 in peripheral neutrophils was detected using western blot. Compared with the control group, citH3 level in the peripheral neutrophils from tMCAO mice was significantly up-regulated in the OGD/R-treated neuron co-culture group (Fig. 6F-G). Then, we transfected small interfering RNAs (siRNAs) into neurons to decrease the CIRP expression (Fig. 6H) and found that CIRP downregulation in OGD/R treated neurons significantly compromised its effect on NETs formation in peripheral neutrophils isolated from tMCAO mice after 6 hours of co-culture (Fig. 6I-J). In conclusion, these results suggested that the OGD/R-treated primary neurons is able to induce NETs formation by releasing CIRP in vitro.

**Figure 7.**
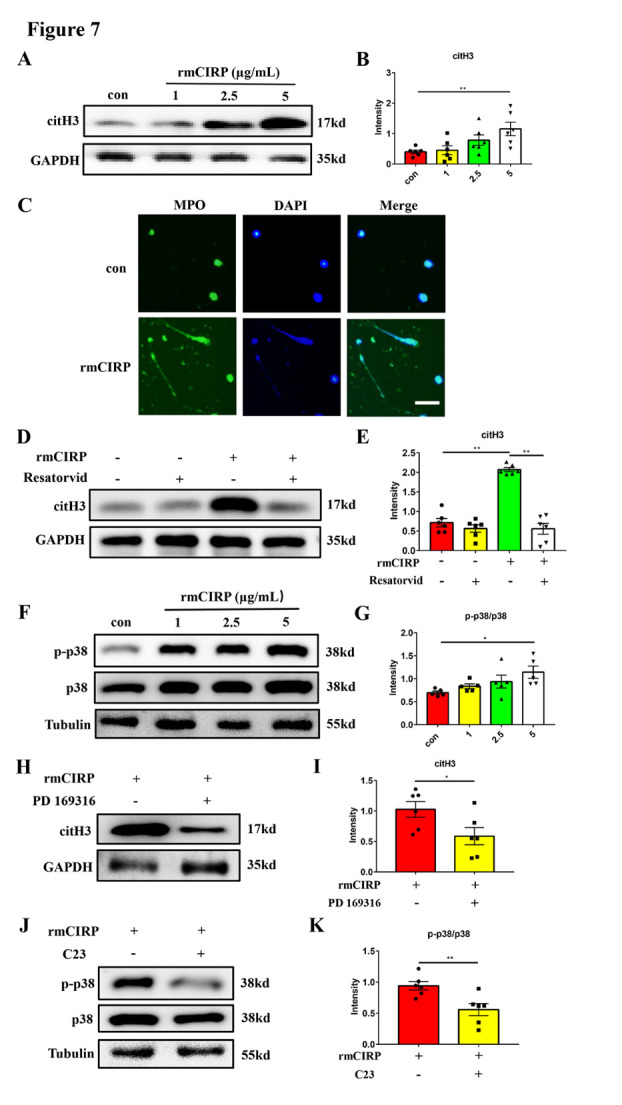
**CIRP induces NETs formation through the TLR4/p38 signaling pathway**. (A and B) Western blot analysis and statistical results of citH3 expression in neutrophils treated with different doses of rmCIRP (1, 2.5, 5 µg/mL) for 6 hours (n = 6/group). Protein levels were normalized to the GAPDH signal. (**C**) Representative images of MPO (green) staining of neutrophils treated with 5 µg/mL rmCIRP for 6 hours. DAPI (blue) was used to stain nuclei. Scar bar: 50 μm. (D and E) Western blot analysis and statistical results of citH3 expression in neutrophils treated with rmCIRP (5 µg/mL) and TLR4 inhibitor Resatorvid (100 nM) for 6 hours (n = 6/group). Protein levels were normalized to the GAPDH signal. (F and G) Western blot analysis and statistical results of p-p38 and p38 expression in neutrophils treated with different doses of rmCIRP (1, 2.5, 5 µg/mL) for 6 hours (n = 5/group). Protein levels were normalized to the Tubulin signal. (H and I) Western blot analysis and statistical results of citH3 expression in neutrophils treated with rmCIRP (5 µg/mL) and p38 inhibitor PD169316 (10 µM) for 6 hours (n = 6/group). Protein levels were normalized to the GAPDH signal. (J and K) Western blot analysis and statistical results of p-p38 and p38 expression in neutrophils treated with rmCIRP (5 µg/mL) and CIRP inhibitor C23 (0.3 µg/mL) for 6 hours (n = 6/group). Protein levels were normalized to the Tubulin signal. *p < 0.05, **p < 0.01. rmCIRP, recombinant murine CIRP; TLR4, toll-like receptor 4; citH3, citrullinated histone H3; MPO, myeloperoxidase.

**Figure 8.**
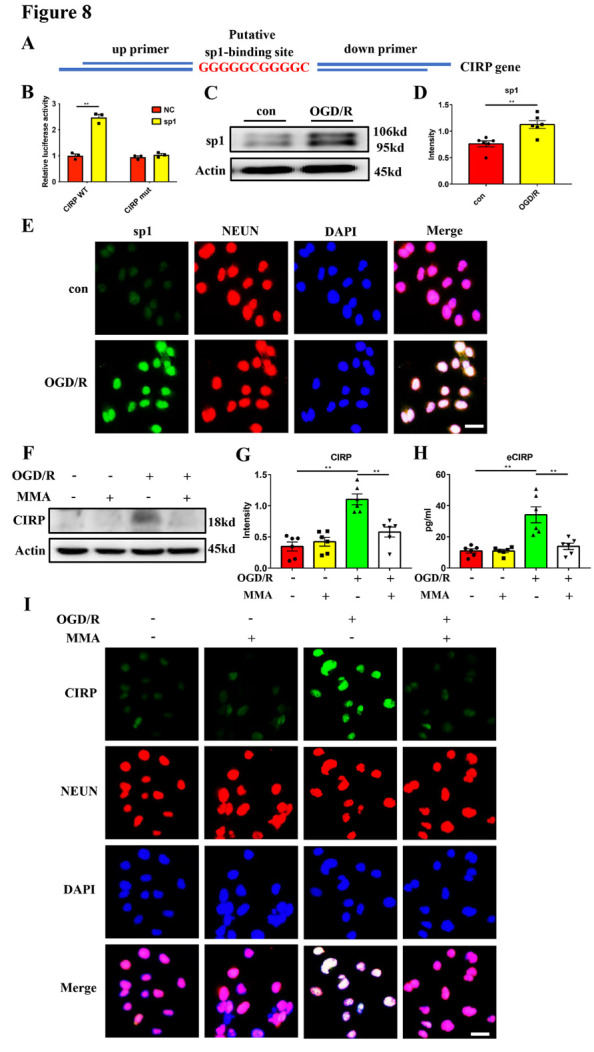
**Transcription factor sp1 promotes CIRP expression in Oxygen-glucose deprivation/reperfusion treated primary neurons**. (**A**) The predicted binding site between sp1 and CIRP promotor by JASPAR (http://jaspar.genereg.net). (**B**) The dual luciferase assay in 293T cells co-transfected with sp1 plasmids and PGL3.basic plasmids with wild-type CIRP promoter, or mutant sequence (n = 3/group). (C and D) Western blot analysis and statistical results of sp1 expression in primary neurons exposed to 6 hours of OGD/R stimulus (n = 6/group). Protein levels were normalized to the actin signal. con, control group in which neurons were not exposed to OGD/R stimulus. (**E**) Representative images of sp1 (green) and NEUN (red) staining of primary neurons treated with 6 hours of OGD/R. DAPI (blue) was used to stain nuclei. Scale bar: 25 μm. (F and G) Western blot analysis and statistical results of the CIRP expression in primary neurons pretreated with sp1 inhibitor, MMA, after 6 hours of OGD/R (n = 6/group). Protein levels were normalized to the actin signal. (**H**) Quantification of CIRP concentration in the supernatant of MMA-pretreated primary neurons after 6 hours of OGD/R (n = 6/group). (**I**) Representative images of CIRP (green) and NEUN (red) staining of MMA-pretreated primary neurons after 6 hours of OGD/R. DAPI (blue) was used to stain nuclei. The MMA was added at the concentration of 300 nM. Scale bar: 25 μm. **p < 0.01. sp1, specificity protein 1; CIRP, cold-inducible RNA-binding protein; OGD/R, oxygen-glucose deprivation/reperfusion.

### CIRP induces NETs formation via TLR4/p38 signaling pathway

We further stimulated neutrophils with different concentrations of rmCIRP (1, 2.5, 5 µg/mL) for 6 hours to investigate its effect on NETs formation. The results showed that rmCIRP stimulation increased the expression of NETs in a dose-dependent manner (Fig. 7A-B). Immunofluorescence staining also revealed the long tail of neutrophils 6 hours after rmCIRP treatment, which is the typical form of neutrophils in NETs formation (Fig. 7C, and Supplementary Fig. 5). These results strongly demonstrated that rmCIRP could induce NETs formation in vitro.

Previous studies have reported that CIRP has a high affinity with the toll-like receptor 4 (TLR4) and triggering receptor expressed on myeloid cells 1 (TREM1) [26, 27]. To identified which receptor mediating the effect of CIRP on NETs formation in ischemic stroke, we detected the expression of TLR4 and TREM1 in peripheral neutrophils isolated from tMCAO mice (24 hours) and found that the TLR4 expression was significantly elevated, whereas TREM1 was only increased slightly (Supplementary Fig. 6A-B). We further found that Resatorvid, the TLR4 inhibitor, effectively alleviated citH3 expression in neutrophils treated with rmCIRP for 6 hours (Fig. 7D-E), while Resatorvid (100 nM) had no significant toxicity in neutrophils (Supplementary Fig. 7A). These results suggested that TLR4 participated in the effect of CIRP on NETs formation. It is well established that MAPK pathway mediates TLR4 activation to regulate neutrophil activation [28]. Thus, we investigated whether CIRP can similarly activate MAPK pathway via TLR4 to promote NETs formation. Western blot showed that the p-p38/p38 ratio was elevated in neutrophils treated with rmCIRP for 6 hours in a dose-dependent manner (Fig. 7F-G), while p38 inhibitor PD169316 significantly abrogated the effect of rmCIRP on NETs formation (Fig. 7H-I). PD169316 (10 µM) had no significant toxicity in neutrophils (Supplementary Fig. 7B). CIRP inhibitor C23 also decreased the ratio of p-p38/p38 in neutrophils treated with rmCIRP for 6 hours (Fig. 7J-K). From these results, it can be concluded that TLR4/p38 signaling pathway may be involved in CIRP-induced NETs formation.

**Figure 9.**
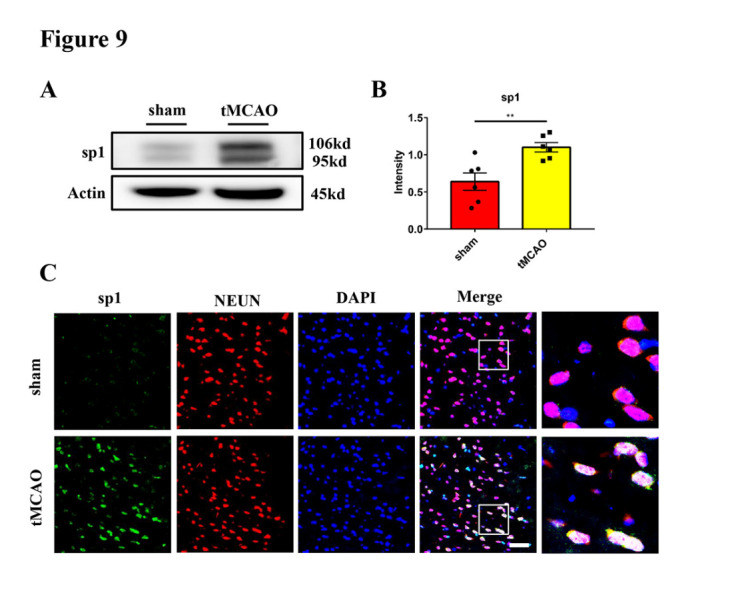
**The expression of sp1 was increased in neurons in transient middle cerebral artery occlusion mice**. (A and B) Western blot analysis and statistical results of sp1 expression in the ischemic penumbra of tMCAO (24 hours) mice (n = 6/group). Protein levels were normalized to the actin signal. (**C**) Representative images of sp1 (green) and NEUN (red) staining of mice brain sections from sham and tMCAO (24 hours) groups. DAPI (blue) was used to stain nuclei. Scale bar: 50 μm. **p < 0.01. sp1, specificity protein 1; tMCAO, transient middle cerebral artery occlusion.

### Transcription factor sp1 promotes CIRP expression in OGD/R-treated primary neurons

Further, we investigated the mechanism of CIRP expression in primary neurons exposed to OGD/R stimulus. Previous studies have reported that under hypoxic conditions, the expression of transcription factor sp1 was increased and sp1 can bind to the CIRP promoter [29]. Then, we posited that sp1 might be involved in the regulation of CIRP expression in OGD/R-treated neurons. Using the bioinformatics software JASPAR (http://jaspar.genereg.net), we successfully predicted the binding site of sp1 and CIRP promoter: GGGGGCGGGGC (Fig. 8A). To further validate their binding, we performed the dual-luciferase assay and found that only CIRP WT and sp1 plasmids co-transfection resulted in the activation of luciferase intensity (Fig. 8B), indicating the participation of sp1 in CIRP transcription. Further, western blot and immunofluorescence staining experiments showed that the expression of sp1 was significantly increased in primary neurons exposed to 6 hours of OGD/R (Fig. 8C-E and Supplementary Fig. 8A). Treatment with MMA, the sp1 specific inhibitor, resulted in a significant reduction of CIRP expression as well as its release from neurons subjected to 6 hours of OGD/R (Fig. 8F-I and Supplementary Fig. 8B). These results indicated that the increased expression of transcription factor sp1 promoted CIRP expression in OGD/R-treated primary neurons.

**Figure 10.**
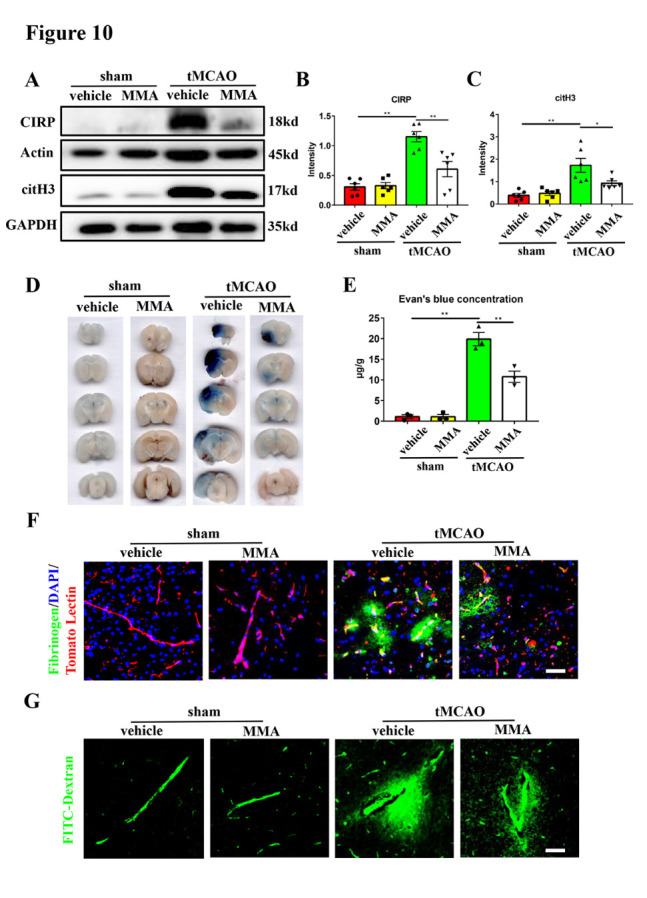
**Inhibition of sp1 can ameliorate NETs formation and brain endothelial barrier disruption in transient middle cerebral artery occlusion mice**. (**A-C**) Western blot analysis and statistical results of the expression of CIRP and citH3 in the ischemic penumbra of sham + vehicle, sham + MMA, tMCAO + vehicle and tMCAO + MMA groups 24 hours after tMCAO (n = 6/group). Protein levels were normalized to the GAPDH signal for citH3 and actin signal for CIRP. MMA was intraperitoneally injected at 250 μg/kg at the start of reperfusion. (**D**) The Evan’s blue extravasation of mice brains in the coronal sections from these 4 groups. (**E**) The Evan’s blue concentration expressed as μg/g of brain tissue (n = 3/group). (**F**) Immunofluorescence staining showing the leakage of fibrinogen (green) in the mice brains from these 4 groups. The blood vessels were stained with Tomato Lectin (red). DAPI (blue) was used to stain nuclei. Scale bar: 50 μm. (**G**) Representative images of extravascular FITC-dextran fluorescence in the mice brains from these 4 groups. Scale bar: 50 μm. *p < 0.05, **p < 0.01. sp1, specificity protein 1; tMCAO, transient middle cerebral artery occlusion; CIRP, cold-inducible RNA-binding protein; citH3, citrullinated histone H3; MMA, mithramycin A.

### Transcription factor sp1 induces neuronal CIRP expression in tMCAO mice

Next, we investigated the role of sp1 in regulating neuronal CIRP expression in the mouse model of tMCAO. First, we found an increased expression of neuronal sp1 in the ischemic penumbra 24 hours after tMCAO (Fig. 9A-C). Next, we intraperitoneally injected the sp1 inhibitor MMA, into the tMCAO mice to suppress its function, immediately after reperfusion. The mice were randomly divided into four groups: sham mice + vehicle injection (sham + vehicle), sham mice + MMA injection (sham + MMA), tMCAO mice + vehicle injection (tMCAO + vehicle), and tMCAO mice + MMA injection (tMCAO + MMA). The brain endothelial barrier leakage was evaluated 24 hours after tMCAO. Compared with the tMCAO + vehicle group, the levels of both CIRP and citH3 were significantly reduced in the tMCAO + MMA group 24 hours after tMCAO (Fig. 10A-C), demonstrating that sp1 inhibition reduced CIRP expression and NETs formation. In addition, the tMCAO + MMA group showed a decreased leakage of Evan’s blue, fibrinogen and FITC-Dextran compared with the tMCAO + vehicle group (Fig. 10D-G). CIRP and NETs expression, as well as Evan’s blue, fibrinogen and FITC-Dextran leakage in the sham + MMA group showed no significant difference with those of the sham + vehicle group (Fig. 10A-G). To conclude, these results strongly indicated that sp1 inhibition can reduce CIRP expression, NETs formation and brain endothelial barrier destruction in tMCAO mice.

### Upregulated CIRP level is associated with cerebral edema in patients with acute ischemic stroke

**Figure 11.**
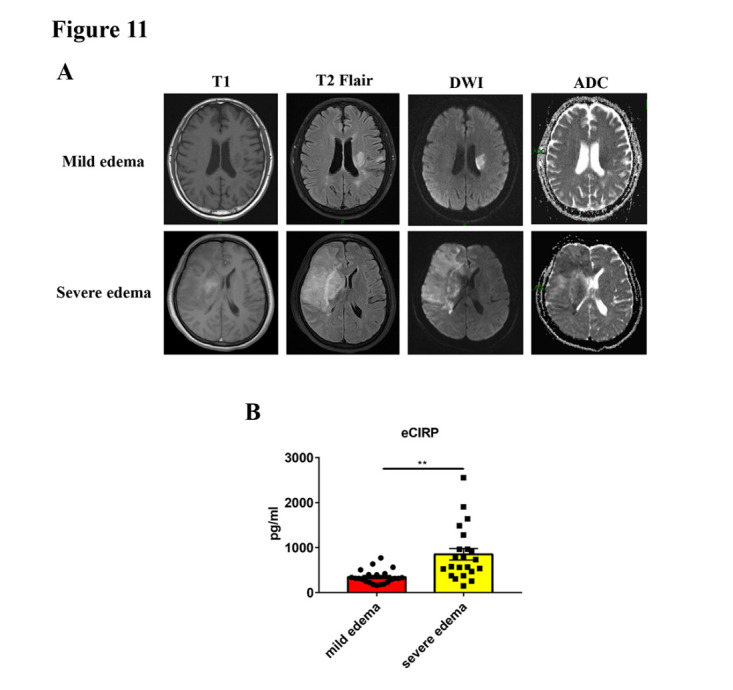
**Upregulated CIRP level is associated with cerebral edema severity in acute ischemic stroke patients**. (**A**) Representative MRI images from acute ischemic stroke patients with mild and severe cerebral edema. (**B**) The serum concentration of CIRP (eCIRP) in the mild and severe cerebral edema groups (n = 22/group). **p < 0.01. CIRP, cold-inducible RNA-binding protein; MRI, magnetic resonance imaging; DWI, diffusion weighted imaging; ADC, apparent diffusion coefficient.

According to previous results, we demonstrated that ischemic neurons could release CIRP to promote neutrophil NETs formation, which subsequently led to brain endothelial barrier disruption and vasogenic edema. Thus, CIRP seems to be an important factor in causing cerebral edema after ischemic stroke. To further investigate the role of CIRP in cerebral edema in clinical practice, 44 patients with acute ischemic stroke recruited from January 1, 2023, to April 30, 2023, at Union Hospital, Tongji Medical College, Huazhong University of Science and Technology, Wuhan, China were divided into the mild and severe cerebral edema groups according to the degree of edema (Fig. 11A). The serum level of CIRP was determined by ELISA. The clinical baseline characteristics were shown in Table 1. We found that the severe cerebral edema group had a higher level of serum CIRP compared with the mild cerebral edema group (Fig. 11B), which indicated that CIRP might be a possible biomarker of cerebral edema progress after acute ischemic stroke.

## DISCUSSION

**Figure 12.**
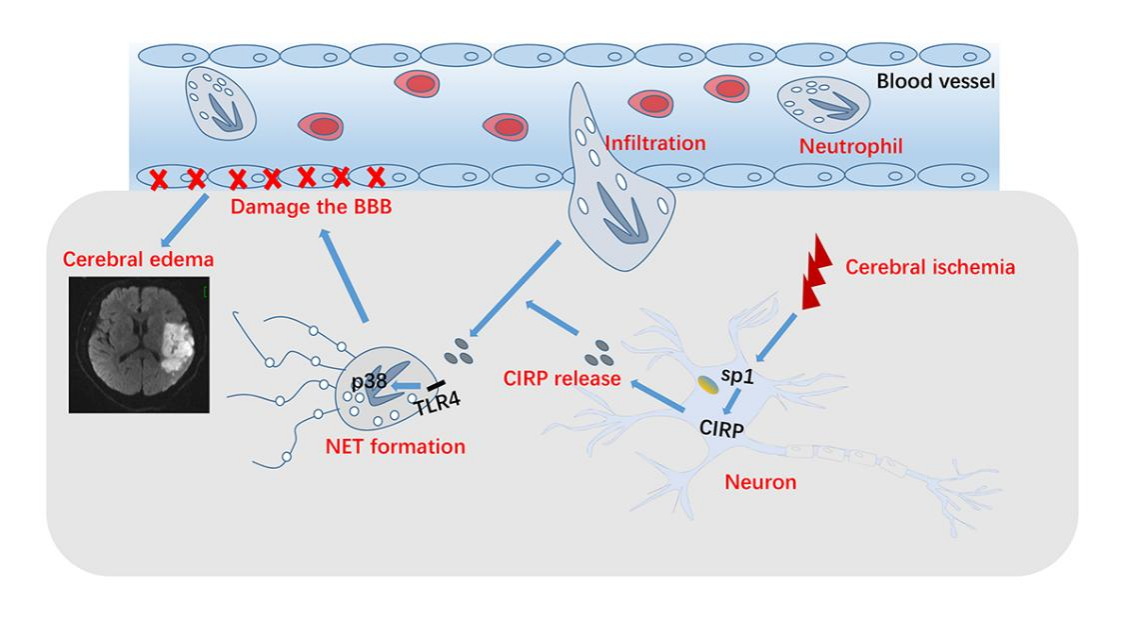
The schematic illustration shows the mechanism of how neurons derived cold-inducible RNA-binding protein promotes NETs formation to exacerbate brain endothelial barrier disruption and cerebral edema after ischemic stroke.

In this study, we first found that neutrophils infiltrating the penumbra form NETs, and degradation of NETs attenuated brain endothelial barrier destruction after ischemic stroke. Then, we further revealed that CIRP released by ischemic neurons induced NETs formation and exacerbated brain endothelial barrier destruction. We also revealed that transcription factor sp1 regulates CIRP expression in neurons and CIRP may promote NETs formation via TLR4/p38 pathway. The results implied that neuronal CIRP mediated neutrophilic NETs-induced brain endothelial barrier disruption and cerebral edema in ischemic stroke, which represents a potential therapeutic target in stroke (Fig. 12).

The inflammatory response mediated by neutrophils infiltrating the brain parenchyma plays a crucial role in brain endothelial barrier destruction after ischemic stroke and NETs formation has been reported to participate in a variety of diseases and amplify the inflammatory response. In our study, we demonstrated that the expression of citH3 started to increase at 6 hours after reperfusion, significantly increasing at 24 hours, and peaking at 3 days in the tMCAO model. Similarly, Kim et al. also reported the occurrence of NETs formation in the mouse model of permanent middle cerebral artery occlusion (pMCAO) and they showed that the citH3 expression increased at 12 hours after pMCAO, peaking at 24 hours, and decreasing after 2 days [30]. The different temporal trends in citH3 expression may be attributed to the different experimental models of ischemic stroke. NETs can damage the endothelium and increase vascular permeability by producing cytotoxic proteases such as elastase, and peroxidase [14]. Therefore, the present study hypothesized that the increased NETs may be responsible for the early brain endothelial barrier destruction after ischemic stroke. We found that DNase I or Cl-amidine treatment significantly attenuated brain endothelial barrier destruction in mice 24 hours after tMCAO. Zhao et al. also showed that NETs released by neutrophils could affect brain endothelial barrier permeability and vascular remodeling 14 days after distal middle cerebral artery embolization in mice [31]. However, they mainly focused on the effect of NETs on neovascularization during the endogenous repair process in ischemic stroke. Our study confirmed that NETs caused brain endothelial barrier destruction even 24 hours after tMCAO. According to previous studies, brain endothelial barrier disruption increased its paracellular permeability, allowed the extravasation of blood components into the brain and resulted in the formation of vasogenic edema especially at 24 hours after MCAO [6], suggesting that NETs also contributes to the brain edema after ischemic stroke.

As a member of DAMPs, CIRP has been proven to induce neutrophils to undergo NETs formation, mediate inflammatory storm, and aggravate sepsis [12]. Moreover, it is reported that CIRP is involved in neuronal apoptosis [32] and microglial inflammation response [33]. Our current study found that the expression of CIRP started to increase at 6 hours after tMCAO, significantly increasing at 24 hours, and peaking at 3 days, which was expressed mainly in neurons and only a few in microglia, astrocytes and endothelial cells in the penumbra. These results were slightly different from Wang’s group. They found that CIRP expression was increased 30 and 48 hours after pMCAO and proved that microglia could express CIRP under OGD condition without reperfusion *in vitro* [34]. Notwithstanding, their results were not contradictory with ours. First, the inconsistent expression trend of CIRP after ischemic stroke may be attributed to different animal models, tMCAO and pMCAO. Second, although Wang’s group demonstrated CIRP expression in microglia *in vitro*, they did not illustrate that neurons do not express CIRP. Third, our results also showed that microglia express CIRP, which is consistent with Wang’s conclusion, but we found that neurons expressed a greater amount of CIRP in the tMCAO model. Most importantly, we proved that specifically downregulating neuronal CIRP could inhibit NETs formation and brain endothelial barrier destruction in tMCAO mice. Significantly, *in vitro* experiments showed that both the expression and release of CIRP were increased in primary neurons after OGD/R treatment. Co-culture of OGD/R-treated primary neurons with neutrophils isolated from tMCAO mice initiated NETs formation and suppression of neuronal CIRP expression abrogated this effect. Although we cannot exclude the potential release of CIRP from microglia, these results indicated that CIRP released from ischemic neurons may be the largest source to induce NETs formation after ischemic stroke. Moreover, we explored how CIRP was secreted and found that compared with the control group, the lysosomal CIRP level was significantly increased 6 hours after OGD/R, indicating the possibility of lysosomal secretion. Further, we demonstrated that CIRP promotes NETs formation through the TLR4/p38 signaling pathway and inhibition of either TLR4 or p38 could significantly abrogate the effect of CIRP on NETs formation.

We further investigated the mechanism of regulating CIRP expression in ischemic neurons and identified sp1 as its transcription factor, which is increased after ischemic stroke and mainly co-localizes with neurons [35]. In addition, sp1 is involved in the pathogenesis of cerebral edema after stroke by regulating Ca-ATP ion channels [35]. In this study, we used the JASPAR website to predict a potential binding site between sp1 and CIRP, and further validated their binding site using dual-luciferase assay. The expression of sp1 was increased in ischemic neurons and inhibition of sp1 could alleviate NETs formation and brain endothelial barrier destruction in tMCAO mice. Furthermore, we found that CIRP can be released from neurons into circulation and the eCIRP level was significantly increased in the peripheral blood of ischemic mice. Considering that NETs play an important role in brain endothelial barrier damage and cerebral edema, CIRP seems to be an important factor to cause cerebral edema after ischemic stroke. In line with this, we confirmed that acute ischemic stroke patients with severe cerebral edema had a higher serum CIRP level compared with the mild cerebral edema group, which perhaps may serve as a predictive marker for cerebral edema progress to guide clinical decision.

However, there are several drawbacks in the current study. First, NETs formation occurs in both the intravascular and brain parenchymal space after ischemic stroke. We didn’t precisely distinguish whether the effect of NETs on brain endothelial barrier is the product of intravascular or parenchymal events. We found that the citH3 level in peripheral neutrophils started to increase at 1 hour after ischemic stroke and reached the peak at 12 hours. Thus, intravascular NETs formation at early time points (1-12 hours) may contribute to the brain endothelial barrier disruption. However, this time peak in the vasculature (12 hours) does not match the peak of brain endothelial barrier disruption (1-3 days). In addition, the proportion of NETs formation was relatively low and only accounted for about 20% of the total peripheral neutrophils [30]. Thus, we speculated that intravascular NETs formation may not be the most important contributor to brain endothelial barrier damage. In contrast, intracranial NETs formation peaks at 1-3 days after ischemic stroke, matching the peak of brain endothelial barrier disruption. Therefore, we assumed that the parenchymal events may be the most predominant in brain endothelial barrier damage. Next, we didn’t precisely distinguish the CIRP role in inducing intravascular or brain parenchymal NETs formation. Due to the disrupted brain endothelial barrier, the released CIRP is also able to enter into the circulation, and thus act on intravascular neutrophils as well. However, the concentration of CIRP should decline from intracranial space to peripheral blood with its diffusion outward. The intracranial CIRP concentration is much higher than the intravascular. Furthermore, the peak of CIRP expression occurs at 1-3 days after tMCAO, which is also the time point of neutrophils infiltrating the cerebral parenchyma. However, the number of neutrophils that undergo NETs formation in the peripheral blood start to decrease at 1-3 days after tMCAO, which suggests that CIRP is mainly interacted with brain parenchymal neutrophils to induce NETs formation. Hence, the more significant role exerted by CIRP might occur when neutrophils have infiltrated the parenchymal tissue.

In conclusion, this study illustrated the role of NETs in brain endothelial barrier damage after ischemic stroke and emphasized CIRP as a possible therapeutic target. We further revealed that transcription factor sp1 is able to regulate CIRP expression in ischemic neurons and CIRP may promote NETs formation in tMCAO mice via the TLR4/p38 signaling pathway, which may also provide insights into stroke treatment. We suggest that CIRP-induced NETs formation is a key target for amelioration of brain endothelial barrier disruption and malignant cerebral edema after ischemic stroke.

### Conclusion

Using a mouse model of tMCAO, our study showed that in response to ischemia-reperfusion stimulus, neurons increased the expression and release of CIRP by up-regulating the transcription factor sp1, which subsequently promoted NETs formation in neutrophils infiltrating the ischemic penumbra, leading to brain endothelial barrier destruction. The finding highlights the potential role of CIRP in brain endothelial barrier protection and acute ischemic stroke treatment.

## Supplementary Materials

The Supplementary data can be found online at: www.aginganddisease.org/EN/10.14336/AD.2024.0204-1. All data generated during the present study can be provided by the corresponding author for a reasonable request.


