# Supplementary material for: Neuron Derived Cold-Inducible RNA-Binding Protein Promotes NETs Formation to Exacerbate Brain Endothelial Barrier Disruption after Ischemic Stroke
Source: Aging Dis. 2024 Feb 4;16(1):520–39. doi: 10.14336/AD.2024.0204-1 (PMC11745456; doi:10.14336/AD.2024.0204-1)
Supplement: Supplementary file 1 [file AD-16-1-520-s.pdf]

SUPPLEMENTARY DATA

Neuron Derived Cold-Inducible RNA-Binding Protein Promotes NETs Formation to Exacerbate Brain Endothelial Barrier Disruption after Ischemic Stroke

Zhifang Li, Shuai Sun, Qinghui Xiao, Senwei Tan, Huijuan Jin, Bo Hu

© 2024. Li Z et al. Published online at http://www.aginganddisease.org/EN/10.14336/AD.2024.0204-1

SUPPLEMENTARY DATA

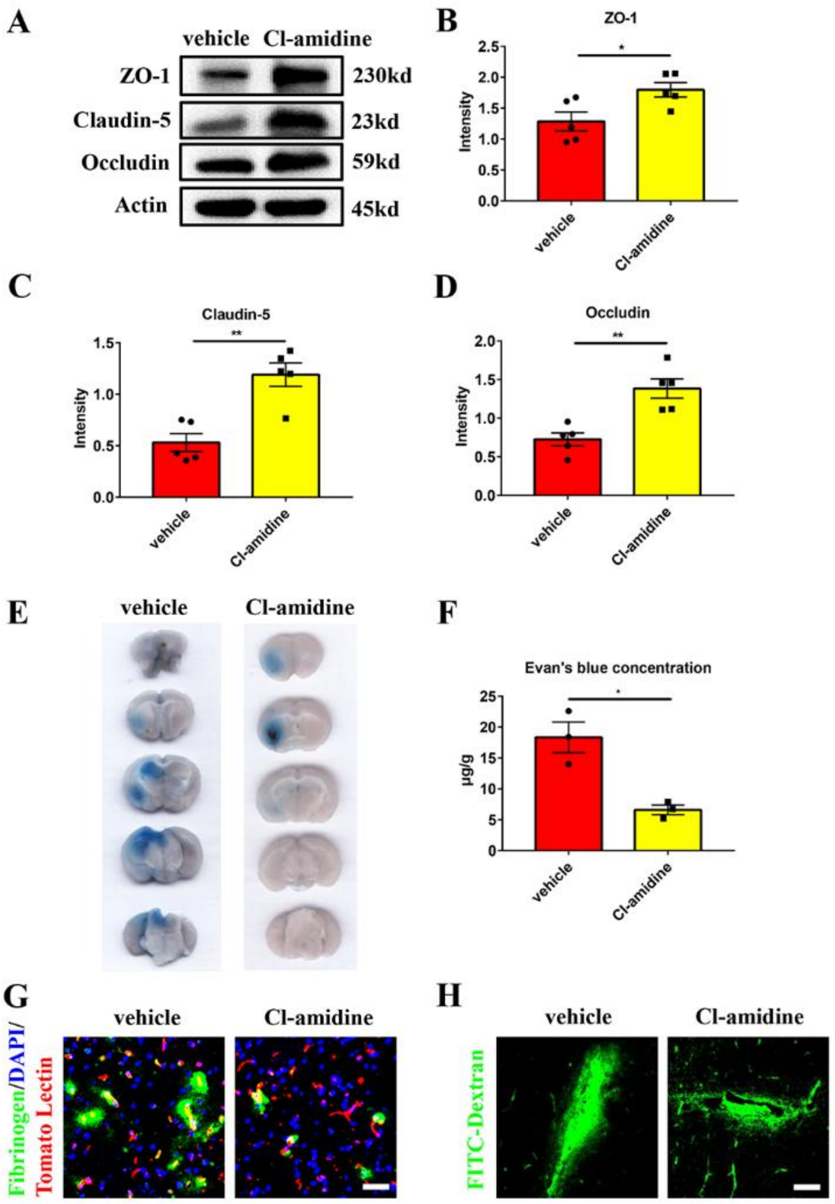

Supplementary Figure 1. PAD inhibitor Cl-amidine treatment can ameliorate brain endothelial barrier destruction in transient middle cerebral artery occlusion mice. A-D: Western blot analysis and statistical results of the expression of ZO-1, claudin-5, and occludin in the ischemic penumbra 24 hours after tMCAO (n = 5/group). Protein levels were normalized to the actin signal. E: The Evan's blue extravasation of mice brains in the coronal sections from the vehicle and Cl-amidine groups. F: The Evan's blue concentration expressed as µg/g of brain tissue (n = 3/group). G: Immunofluorescence staining showing the leakage of fibrinogen (green) in the tMCAO mice brains from the vehicle and Cl-amidine groups. The blood vessels were stained with Tomato Lectin (red). DAPI (blue) was used to stain nuclei. Scale bar: 50 µm. H: Representative images of extravascular FITC-dextran fluorescence in the mice brains from the vehicle and Cl-amidine groups. Scale bar: 50 µm. *$p < 0.05$, **$p < 0.01$. PAD, Peptidylarginine deiminase; tMCAO, transient middle cerebral artery occlusion; ZO-1, zona occludens-1; citH3, citrullinated histone H3.

© 2024. Li Z et al. Published online at http://www.aginganddisease.org/EN/10.14336/AD.2024.0204-1

SUPPLEMENTARY DATA

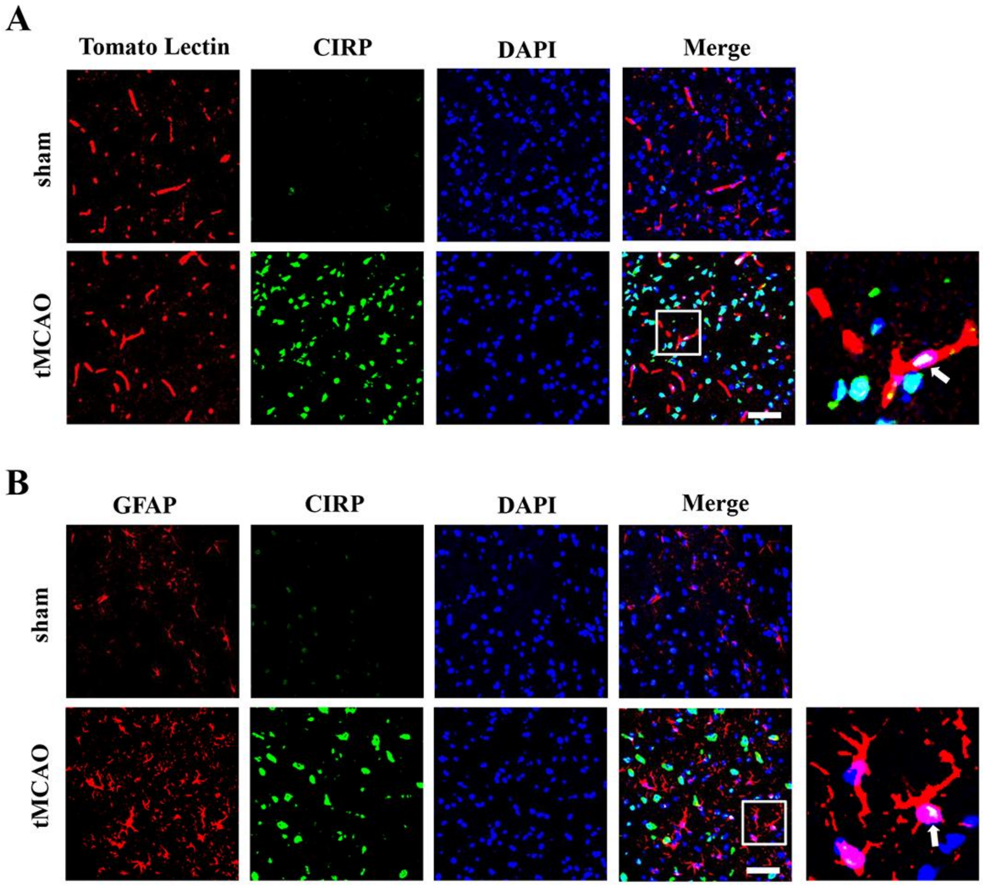

Supplementary Figure 2. The expression of CIRP in the endothelial cells and astrocytes 24 hours after transient middle cerebral artery occlusion. A: Representative images of CIRP (green) and Tomato Lectin (red) staining of brain sections from sham and tMCAO (24 hours) mice. DAPI (blue) was used to stain nuclei. The white arrow indicates the co-localization of CIRP and Tomato Lectin. Scale bar: 50 μm. B: Representative images of CIRP (green) and GFAP (red) staining of brain sections from sham and tMCAO (24 hours) mice. DAPI (blue) was used to stain nuclei. The white arrow indicates the co-localization of CIRP and GFAP. Scale bar: 50 μm. CIRP, cold-inducible RNA-binding protein; tMCAO, transient middle cerebral artery occlusion.

© 2024. Li Z et al. Published online at http://www.aginganddisease.org/EN/10.14336/AD.2024.0204-1

SUPPLEMENTARY DATA

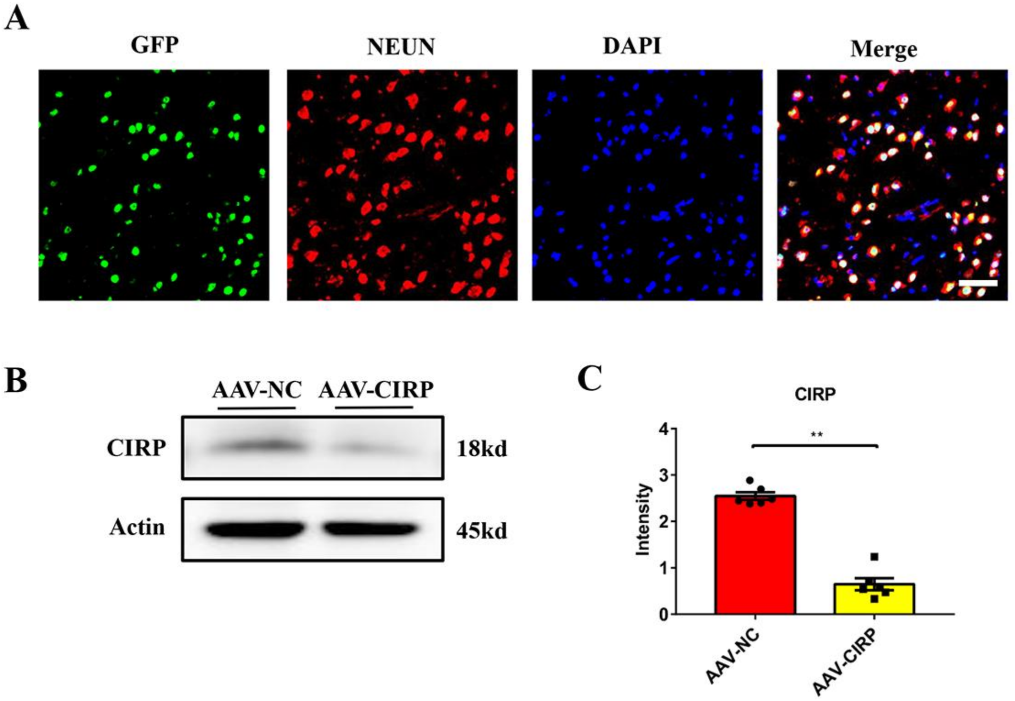

Supplementary Figure 3. Mice was injected with AAV to specifically downregulate neuronal CIRP. A: Representative images of NEUN (red) staining of brain sections from AAV infected mice. DAPI (blue) was used to stain nuclei. Scale bar: 50 μm. B and C: Western blot analysis and statistical results showing cerebral CIRP expression 4 weeks after AAV infection (n = 6/group). Protein levels were normalized to the actin signal. ***p* < 0.01. CIRP, cold-inducible RNA-binding protein; AAV, adeno-associated virus.

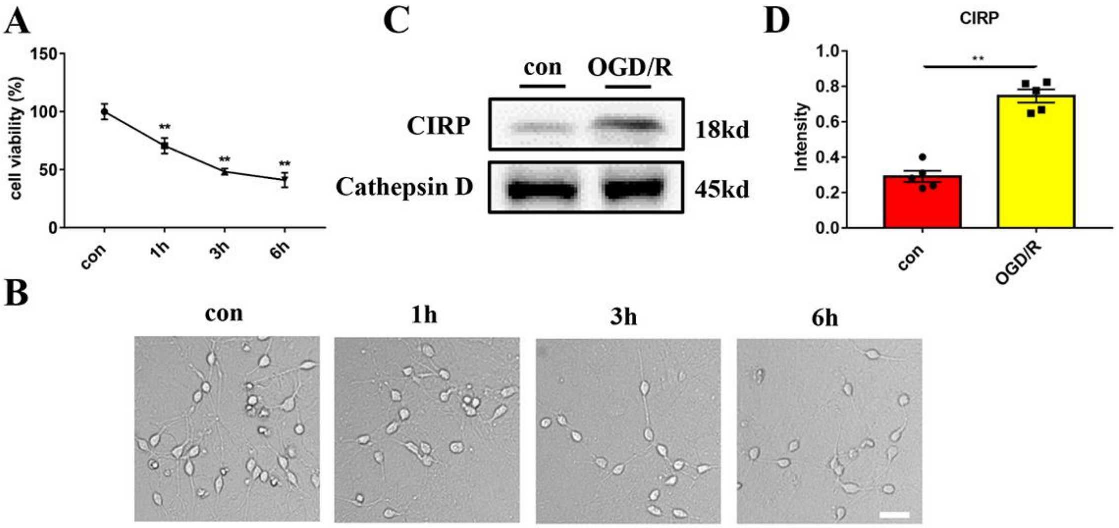

Supplementary Figure 4. **The effect of Oxygen-glucose deprivation/reperfusion stimulus on primary neurons.** A: The CCK-8 assay showing primary neuron viability after different times of OGD/R stimulus (n = 6/group). B: The bright field images showing the morphological changes of primary neuron after different times of OGD/R treatment. Scale bar: 25 μm. C and D: Western blot analysis and statistical results showing lysosomal CIRP expression in primary neurons 6 hours after OGD/R (n = 5/group). Protein levels were normalized to the Cathepsin D signal. ***p* < 0.01. CCK-8, cell counting kit-8; CIRP, cold-inducible RNA-binding protein; OGD/R, oxygen-glucose deprivation/reperfusion.

© 2024. Li Z et al. Published online at http://www.aginganddisease.org/EN/10.14336/AD.2024.0204-1

SUPPLEMENTARY DATA

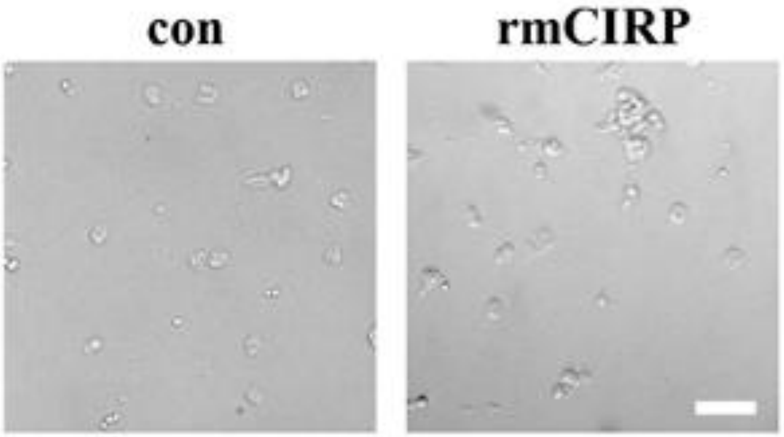

Supplementary Figure 5. The bright field images showing the morphological changes of neutrophils after treatment of rmCIRP (5 µg/mL) for 6 hours. Scale bar: 50 µm.

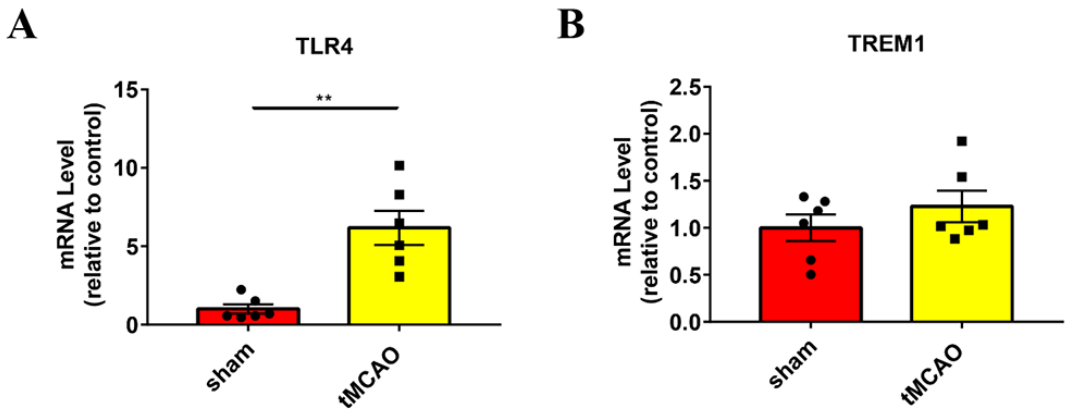

Supplementary Figure 6. The expression of TLR4 and TREM1 in peripheral neutrophils isolated from transient middle cerebral artery occlusion mice. A and B: Quantification of TLR4 and TREM1 mRNA expression in the peripheral neutrophils isolated from sham and tMCAO (24 hours) mice (n = 6/group). TLR4, toll-like receptor 4; TREM1, triggering receptor expressed on myeloid cells 1; tMCAO, transient middle cerebral artery occlusion.

© 2024. Li Z et al. Published online at http://www.aginganddisease.org/EN/10.14336/AD.2024.0204-1

SUPPLEMENTARY DATA

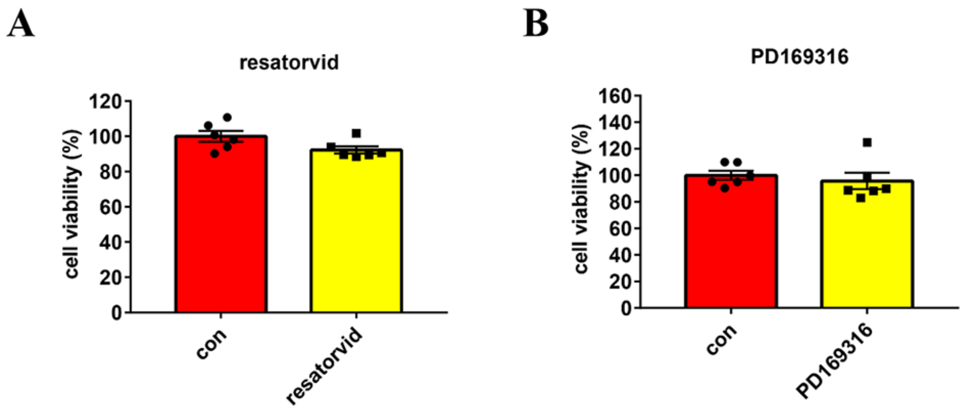

Supplementary Figure 7. A and B: The CCK-8 assay showing neutrophils viability after treatment of resatorvid (100 nM) and PD169316 (10 μM) (n = 6/group). CCK-8, cell counting kit-8.

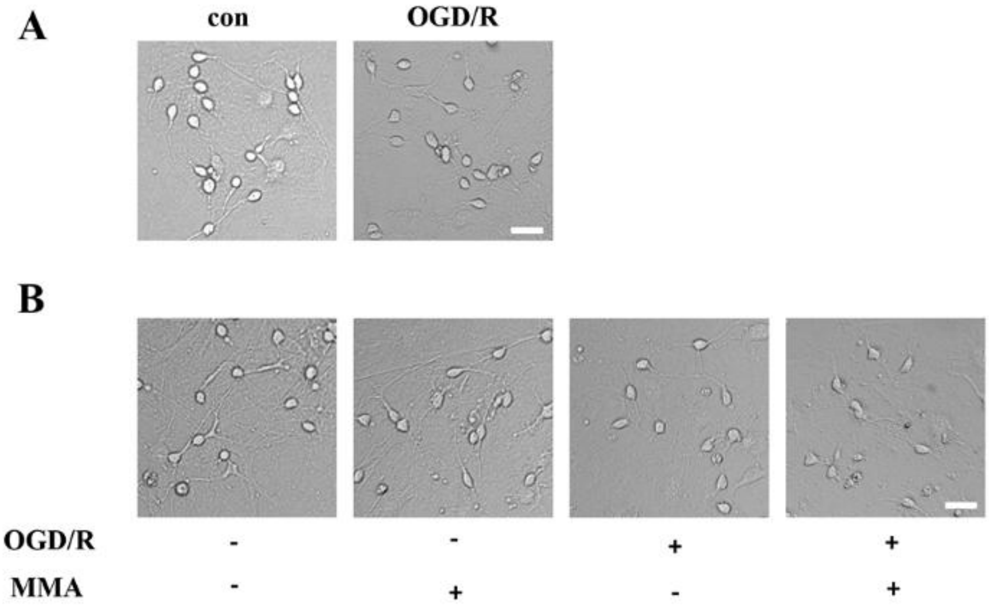

Supplementary Figure 8. A: The bright field images showing the morphological changes of primary neurons after 6 hours of OGD/R stimulus. Scale bar: 25 μm. B: The bright field images showing the morphological changes of primary neurons after 6 hours of OGD/R stimulus and MMA treatment (300 nM). Scale bar: 25 μm. OGD/R, oxygen-glucose deprivation/reperfusion; MMA, mithramycin A.

© 2024. Li Z et al. Published online at http://www.aginganddisease.org/EN/10.14336/AD.2024.0204-1
